# Characterization and the first complete genome sequence of a novel strain of *Bergeyella porcorum* isolated from pigs in China

**DOI:** 10.1186/s12866-024-03366-6

**Published:** 2024-06-17

**Authors:** Gang Liu, Chao Chen, Zhikang Jiang, Yu Liu, Xianwen Wang, Lei Qiao, Kang Liu, Xianjie Han

**Affiliations:** College of Veterinary Medicine, Qingdao Agricultural University, Qingdao, 266109 Shandong China

**Keywords:** 16S rRNA, *Bergeyella porcorum*, Identification, Genome sequencing, Comparative genomics

## Abstract

**Background:**

*Bergeyella porcorum* is a newly identified bacterium that has an ambiguous relationship with pneumonia in pigs. However, few studies have adequately characterized this species.

**Results:**

In this study, we analyzed the morphological, physiological, and genomic characteristics of the newly identified *B. porcorum* sp. nov. strain QD2021 isolated from pigs. The complete genome sequence of the *B. porcorum* QD2021 strain consists of a single circular chromosome (2,271,736 bp, 38.51% G + C content), which encodes 2,578 genes. One plasmid with a size of 70,040 bp was detected. A total of 121 scattered repeat sequences, 319 tandem repeat sequences, 4 genomic islands, 5 prophages, 3 CRISPR sequences, and 51 ncRNAs were predicted. The coding genes of the *B. porcorum* genome were successfully annotated across eight databases (NR, GO, KEGG, COG, TCDB, Pfam, Swiss-Prot and CAZy) and four pathogenicity-related databases (PHI, CARD, VFDB and ARDB). In addition, a comparative genome analysis was performed to explore the evolutionary relationships of *B. porcorum* QD2021.

**Conclusions:**

To our knowledge, this is the first study to provide fundamental phenotypic and whole-genome sequences for *B. porcorum*. Our results extensively expand the current knowledge and could serve as a valuable genomic resource for future research on *B. porcorum*.

**Supplementary Information:**

The online version contains supplementary material available at 10.1186/s12866-024-03366-6.

## Background

*Bergeyella* spp. are nonfermenting gram-negative bacilli that belong to the *Flavobacteriaceae* family and were first described in 1994 [1]. Presently, *B. zoohelcum*, *B. porcorum*, and *B. cardium* have been described in the genus *Bergeyella* [2–4], but only *B. zoohelcum* and *B*. *porcorum* were correctly named according to their taxonomic status (validly published under the ICNP) (https://www.bacterio.net). *B. zoohelcum* is the most commonly identified component of the normal oral flora of animals and is considered an uncommon zoonotic pathogen typically associated with cat or dog bites, resulting in cellulitis, leg abscess, septicemia, tenosynovitis, pneumonia, meningitis, and liver cirrhosis [4]. In contrast, infections caused by other *Bergeyella* spp. have rarely been reported. In 2014, the first case of infective endocarditis caused by *B. cardium* sp. nov. was reported, and *B. cardium* infection is becoming increasingly common [2]. Considering the importance of *B. cardium* in disease pathogenesis, the fundamental phenotypic and genomic information for *B. cardium* was recently studied [5].

In 2016, L. Zamora described a new species of the genus *Bergeyella*, which was named *B. porcorum* sp. nov [3]. To date, *B. porcorum* has rarely been reported, and there is no available complete genome sequence of *B. porcorum.* In this study, we report the first whole genome sequence of *B. porcorum* and genomic analyses, which provide new insights into the biology of *B. porcorum.*

## Materials and methods

### Bacterial isolation

Samples were collected from the dissected lungs of dead pigs with pneumonia at a pig farm in Shandong Province. Lung swabs were plated on blood agar plates and incubated at 37 °C for 24 to 48 h under aerobic and anaerobic conditions. Colonies were selected and purified using blood agar and then stored at -80 °C in Luria–Bertani broth containing 15% glycerol for further analysis.

### Characterization of bacterial isolates

The morphological characteristics of the isolated bacteria were determined by Gram staining, and different biochemical tests were performed for the identification of the bacterial isolates. The principal tests used for this purpose included urease, ornithine decarboxylase, β-galactosidase, catalase, phenylalanine deaminase, arginine dihydratase, sucrose, glucose, sorbitol, mannitol, maltose, ribose, arabinose, tryptophan depletion, pyruvate, and hydrogen sulfide production. Briefly, the fresh culture was inoculated into commercial microtubes (Binhe Microbiological Regent Co., Ltd., Hangzhou, China) for 48 h at 37 °C, after which the reaction results were observed and analyzed according to the manufacturer’s instructions. Moreover, the morphologies of the isolates were further observed by scanning electron microscopy (SEM), and their growth was also tested on MacConkey agar (Haibo Biotechnology, Qingdao, China).

### Phylogenetic analysis

Strains were grown aerobically in 30 mL of brain heart infusion broth (Haibo Biotechnology, Qingdao, China) at 37 °C with shaking at 225 rpm. For preliminary identification, a small fragment of the 16S rRNA gene was amplified using the universal primer set (27 F 5′-AGAGTTTGATCCTGGCTCAG-3′; 1541R 5′-AAGGAGGTGATCCAGCCGCA-3′) [6]. The PCR cycling conditions were as follows: initial denaturation at 95 °C for 5 min; 30 cycles of 95 °C for 15 s, 55 °C for 15 s, and 72 °C for 90 s; and a final elongation at 72 °C for 7 min (GeneAmp PCR System 2700; Applied Biosystems). The amplified products were confirmed by 1% (w/v) agarose gel electrophoresis and then sequenced at Sangon Biotech Co., Ltd. (Shanghai, China). Spliced sequences were compared with online data in the NCBI database (http://www.ncbi.nlm.nih.gov), and multiple sequence alignments were conducted using the MUSCLE (V5.1.0) program [7]. Subsequently, the phylogenetic tree was constructed using the maximum likelihood method in MEGA 7.0 [8].

### Antimicrobial susceptibility testing

The minimum inhibitory concentrations (MICs) of the isolated strains were determined by the broth microdilution method, which employs the following antimicrobial agents: erythromycin (ERY), tetracycline (TET), streptomycin (STR), sulfaisoxazole (SF), timicosin (TIM), zaithromycin (ZAI), penicillin (PEN), enrofloxacin (ENR), and ciprofloxacin (CIP). *E. coli* ATCC 25922 was used as the control strain following the EUCAST guidelines (EUCAST, 2021).

### DNA extraction

Genomic DNA was extracted from the isolated strain for whole-genome sequencing using the TIANamp Bacteria DNA Kit (TIANGEN BIOTECH, Beijing, China). The quality of the products was estimated using a Qubit 2.0 fluorometer (Thermo Scientific, Waltham, MA, USA) and a NanoDrop 2000 spectrophotometer (Thermo Scientific, Waltham, MA, USA), after which the products were subjected to 1% (w/v) agarose gel electrophoresis. To obtain a highly accurate assembly and annotation, a combination of the Illumina NovaSeq PE150 and PacBio Sequel platforms was used for the sequencing of the complete genome of *B. porcurum* at Beijing Novogene Bioinformatics Technology Co., Ltd. (Beijing, China).

### Genome sequencing and assembly

For Illumina sequencing, the qualified DNA was randomly broken into 350 bp fragments with Covaris ultrasonic processors, and the Illumina library was prepared via end repair and the addition of an A tail and sequencing adaptors via an NEBNext® Ultra™ DNA Library Prep Kit (NEB, Ipswich, Ipswich, MA, USA). Next, we used a Qubit 2.0 fluorometer (Thermo Fisher Scientific; Waltham, MA, USA) for initial quantification and then used an Agilent 2100 (Agilent, Santa Clara, California, USA) to confirm that the insert size of the fragments met expectations. Subsequently, the prepared 350 bp library was sequenced on a second-generation Illumina PE150 system. Sequence reads were filtered to exclude adapter and low-quality sequences using FASTP (v0.23.0) with default parameters. All of the results obtained from the Illumina PE150 system were used as the survey for the PacBio third-generation sequencing and to correct preliminary assembly results.

Another part of the DNA sample was broken into 10 kb fragments by Covaris g-TUBE (Covaris, Newtown, Connecticut, USA), and hairpin-type connectors were attached to both ends of the DNA fragments. Next, the fragments were purified with AMpure PB magnetic beads (PacBio, Silicon Valley, California, USA) and screened through a BluePippin instrument (BluePippin, Oakland, California, USA). After that, the genome of *B. porcurum* in the 10 Kb SMRTbell libraries was quantified by a Qubit 2.0 fluorometer, the insert fragment size was detected via an Agilent 2100, and the sequences were subsequently sequenced via the PacBio Sequel platform.

The obtained raw reads were first filtered (˂500 bp) to obtain clean data. According to the automatic error correction function of SMRT LINK v5.0.1 software (https://www.pacb.com/support/software-downloads/) [9, 10], long reads longer than 6,000 bp were selected as the seed sequence, and the remaining shorter reads were aligned to the seed sequence by Blasr (v1.3.1). In addition, the preliminary assembly results were polished using an arrow algorithm (https://github.com/PacificBiosciences/GenomicConsensus), followed by correction with Illumina data by bwa [11]. Furthermore, the result was filtered with a base minimum mass value of 20, a minimum read depth of 4 and a maximum read depth of 1,000. Based on the overlap between the head and the tail, we confirmed whether the chromosomal sequence formed a circle or not, then corrected the initial site by blast with the DNAa database. At last, the chromosome and plasmid sequences were screened by blast with the plasmid database. The genome obtained from the PacBio third-generation sequencing system was used for all of the further analyses.

### Genome annotation and bioinformatic analysis

Coding genes of the sequenced genome were identified by GeneMarkS v4.17 (http://opal.biology.gatech.edu/GeneMark/) software. The interspersed repetitive sequences and the tandem repeats were analyzed using RepeatMasker v4.1.6 (http://www.repeatmasker.org/) and Tandem Repeats Finder (v4.07b), respectively [12, 13]. The tRNAs and ribosomal RNAs (rRNAs) were predicted by tRNAscan-SE (v1.3.1) [14] and rRNAmmer24 (v1.2) [15], respectively. The genes for small RNAs (sRNAs), small nuclear RNAs (snRNAs) and microRNAs (miRNAs) were searched against the Rfam database and confirmed by cmsearch (v1.1rc4) [16, 17]. Furthermore, genomic islands (GIs) and transposons were predicted by the IslandPath-DIOMB (v0.2) and transposonPSI programs, respectively [18, 19]. PhiSpy (v2.3) and CRISPRdigger (v1.0) were used for prophage prediction and CRISPR identification, respectively [20].

### Gene function

Multiple complementary databases, including the Nonredundant Protein Database (NR) [21], Gene Ontology database (GO) [22], Kyoto Encyclopedia of Genes and Genomes (KEGG) [23], Clusters of Orthologous Groups (COG) [24], Transporter Classification Database (TCDB) [25], Pfam, Swiss-Prot [26], and Carbohydrate-Active enZYmes Database (CAZy) [27] were used to predict gene functions. A whole-genome BLAST search with the parameters “E-value ˂ 1e-5, minimal alignment length percentage ˃ 40%” was performed against the above databases. Moreover, pathogenicity and drug resistance were analyzed via the Pathogen Host Interactions Database (PHI) [28], Virulence Factor Database (VFDB) [29], Comprehensive Antibiotic Resistance Database (CARD) [30], and Antibiotic Resistance Genes Database (ARDB) [31]. Subsequently, the secretory proteins were detected in the genome assembly via the SignalP (v4.1) and TMHMM (v2.0c) [32], and the secretion of Type I-VII proteins was predicted via EffectiveT3 (v1.0.1) software [32, 33].

### Comparative genomics analysis

Comparative genomic analyses were performed using the coding sequences and corresponding protein sequences of 16 species downloaded from NCBI, which included 5 *B.* zoohelcum genomes (ATCC43767, CCUG30536, NCTC11660, NCTC11661 and NCTC12929), 3 *B. cardium* genomes (HPQL, 1 and SRR15235668), 2 *Bergeyella* genomes (DRR214960 and SRR15235668), 3 *Weeksellaceae* genomes (*Apibacter mensalis* R-53146, *Weeksella virosa* DSM16922 and *Chishuiella changwenlii* CGMCC 1.12707), and 3 *Riemerella* genomes (*Riemerella anatipestifer* 20190403E1-1, *Riemerella anatipestifer* 20190509E1 and *Riemerella anatipestifer* XG19). First, OrthoFinder v2.5.4 with the parameters (-f: data; -S: diamond; -M: msa; -T: fasttree; and -t: 20) was used to identify the single-copy genes of all the species [34]. Subsequently, all the aligned sequences were concatenated, and the maximum likelihood tree was produced using the RAxML (v8.2.12) software package JTT model with 100 rapid bootstraps in random parsimony (-m PROTGAMMAJTT -p 234 -x 1234 -# 100) [35].

In addition, average nucleotide identity (ANI) and in silico DNA–DNA hybridization (*is*DDH) analyses were performed through the OrthoANIu algorithm (http://www.ezbiocloud.net/tools/orthoaniu) and the Genome-to-Genome Distance Calculator 3.0 (http://ggdc.dsmz.de/ggdc.php), respectively [36, 37]. If the genomic DNA of two organisms reveals a DDH similarity of less than 70% and an ANI similarity of less than 95%, the species are typically considered distinct, and vice versa [38].

## Results

### The genetic identification of the isolate was *Bergeyella Porcorum* sp. nov

**Fig. 1:**
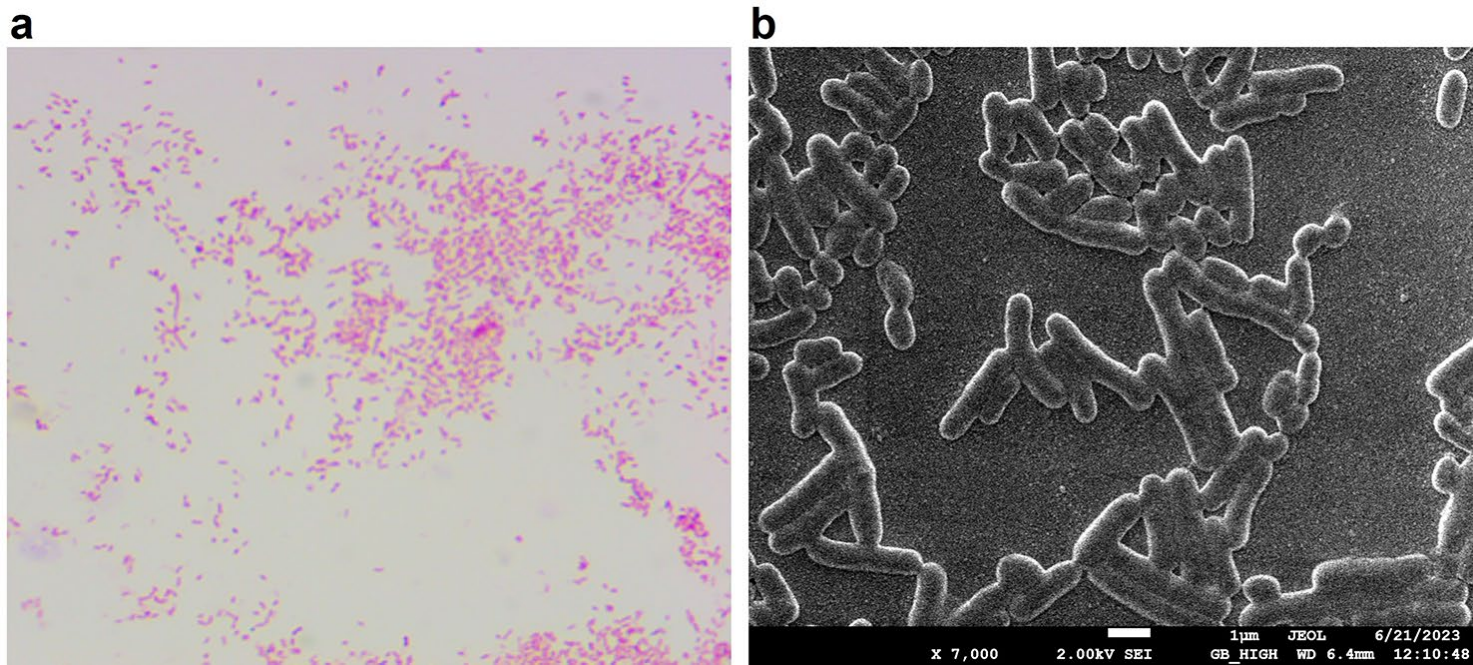
Morphological characterization of *B. porcorum* QD2021. (**a**) Gram staining properties of the QD2021 strain. (**b**) Scanning electron microscopy observation of the bacterial cells

The isolated strain was demonstrated to be a nonfermenting gram-negative bacterium that can grow under anaerobic conditions but did not grow on MacConkey agar plates. Gram staining and SEM observation revealed irregularly rod-shaped bacterial cells (Fig. 1). As indicated by the biochemical assays, this bacterial strain could produce urease and ornithine decarboxylase but not β-galactosidase, arginine dihydratase or catalase. Moreover, the isolated bacteria could not ferment glucose, sucrose, sorbitol, mannitol, maltose, ribose, or arabinose. Additionally, the results were negative for tryptophan, phenylalanine, pyruvate, and hydrogen sulfide.

To accurately identify the pathogenic species, a 1,487 bp 16S rRNA sequence of the isolated strain was amplified and sequenced, and BLASTN was used to determine that the sequence belonged to the genus *Bergeyella*. The 16S rRNA gene of the isolates exhibited the highest identify (> 99%) with that of *B. porcorum*, among which the highest sequence similarity with *B. porcorum* DICM11-00233-2 A was 99.49%, followed by that with *B. porcorum* DICM11-00234-2 A (99.43%), *B. porcorum* 1305-03^T^ (also named CECT9006^T^ and CCUG67887^T^, 99.36%), and *B. porcorum* 612 A-03 (99.35%). In contrast, the 16S rRNA of the isolates exhibited 96–97% identity with *B. zoohelcum*, and 93–94% identity with *B. cardium.* In addition, the PCR-based 16S rRNA BLASTN results were also validated by the predicted genomic 16S rRNA (data not shown).

**Fig. 2:**
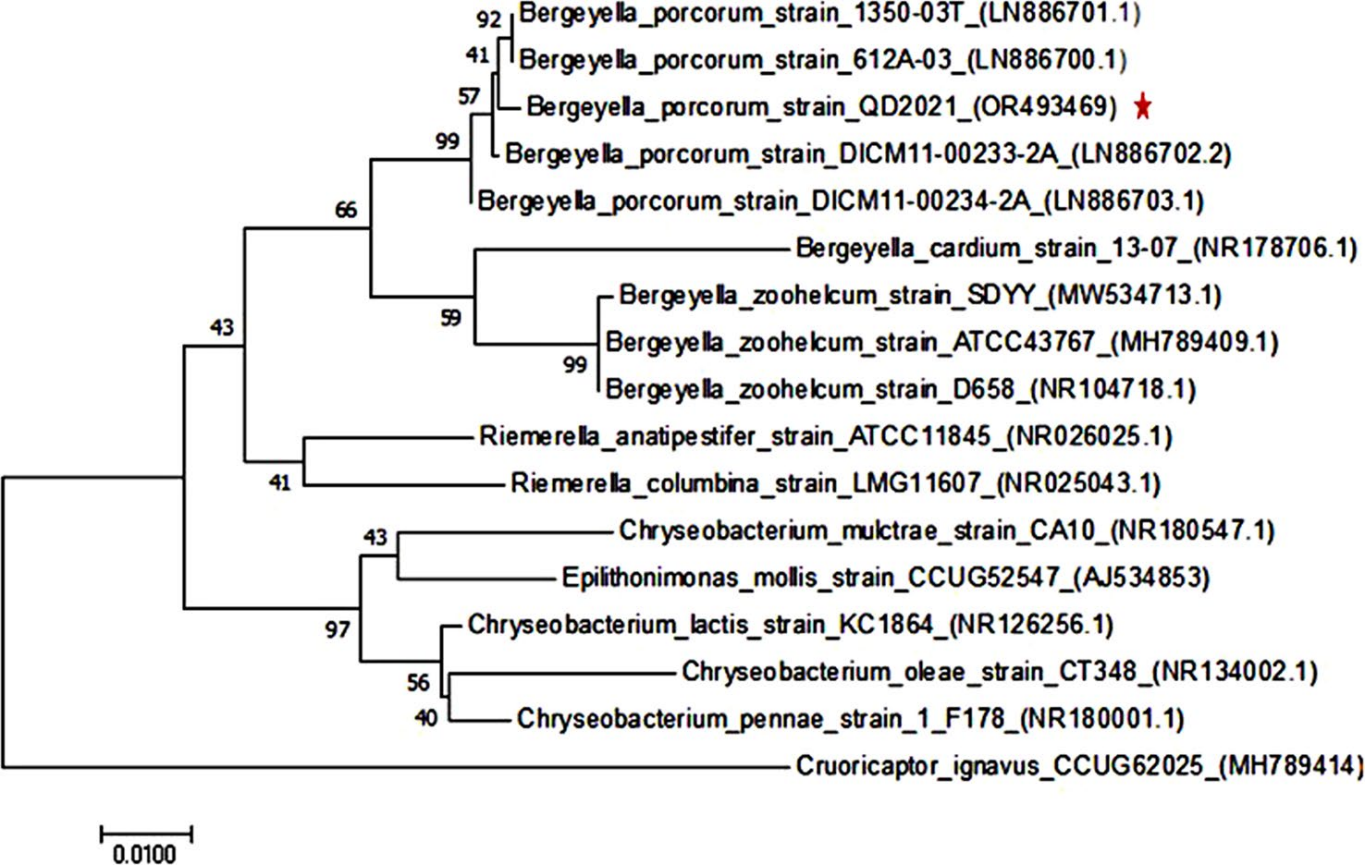
Phylogenetic tree based on 16S rRNA gene sequences of *B. porcorum* QD2021 and closely related species. The QD2021 strain was clustered with *B. porcorum* spp. The phylogenetic tree was created using the maximum likelihoods method. Bootstrap values (1,000 replicates) are shown at the branch points. The scale bar indicates 0.01 nucleotide substitution per nucleotide position. The red star marks the location of strain QD2021

To further verify the nucleotide BLAST results, we constructed a detailed phylogenetic tree, as shown in Fig. 2. The phylogenetic tree revealed that the isolates clustered together with *B. porcorum* strain 1350-03^T^ and *B. porcorum* strain 612 A-03 and formed distinct lineages with *B. zoohelcum* SDYY, *B. zoohelcum* ATCC43767, *B. zoohelcum* D658, and *B. cardium* 13-07. Therefore, the phylogenetic analysis confirmed that the isolated strain in this study was a novel strain of *B. porcorum*; thus, we designated it *B. porcorum* QD2021.

### Antimicrobial susceptibility analysis

The minimum inhibitory concentrations (MICs) of many macrolide antibiotics (erythromycin, tilmicosin, and azithromycin) and aminoglycosides (gentamicin, kanamycin, spectinomycin, and amikacin) were quite high, followed by sulfonamides (sulfamethoxazole and trimethoprim), fluoroquinolones (enrofloxacin and ciprofloxacin) and tetracyclines, with the exception of cephalosporin and levofloxacin (Table 1).

**Table 1:** Minimum inhibitory concentrations of antimicrobial agents against *B. porcorum* QD2021

Antimicrobial agent	MICs (µg/mL)
Erythromycin	256
Tilmicosin	512
Azithromycin	128
Enrofloxacin	64
Ciprofloxacin	32
Levofloxacin	0.25
Streptomycin	32
Gentamicin	256
Kanamycin	64
Spectinomycin	64
Amikacin	32
Sulfamethoxazole	16
Trimethoprim	32
Penicillin	256
Tetracycline	16
Tigecycline	4
Ceftazidime	8
Ceftiofur	4
Cefquinoxime	4
Chloramphenicol	16

### Genomic features of *B. Porcorum* QD2021

**Fig. 3:**
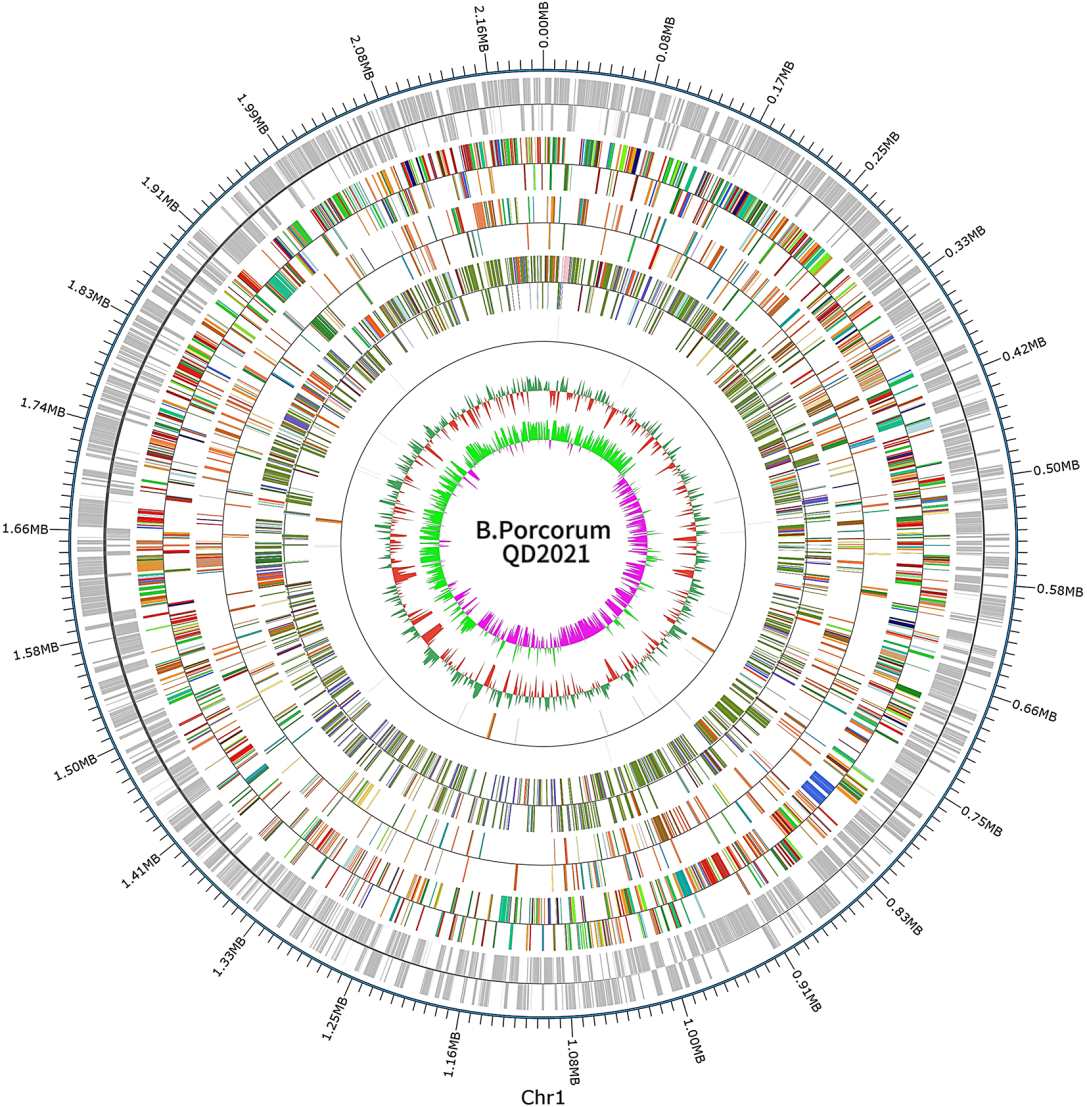
Schematic map of the *B. porcorum* QD2021 genome. The rings from the outer to the inter correspondence corresponded to the following: genome position in kb (ring 1); predicted CDSs on the forward and reverse strands (ring 2); COG annotated genes (ring 3); KEGG annotated genes (ring 4); GO annotated genes (ring 5); ncRNA (ring 6); GC content (ring 7); and GC content deviations from the average (ring 8)

The whole genome of *B. porcorum* QD2021 was further sequenced and analyzed, and the genomic features are described in Table 2; Fig. 3. Briefly, the mean concordance of reads was 0.89, the N50 read length was 487,856 bp, the genome completeness and contamination were 99.99% and 0.06%, respectively. *B. porcorum* QD2021 contained a circular chromosome 2,271,736 bp in length, with a mean GC content of 38.51%, and a total of 1,991,115 bp (87.65%) of predicted coding sequences were identified. The complete genome sequence contained 2,578 predicted coding sequences (CDSs), including 42 tRNAs, 9 rRNAs, and 170,040 bp plasmid pQD2021 (accession number CP136427.1), which had no similarity to known plasmids. However, no sRNAs or miRNAs were predicted.

**Table 2:** Genomic characteristics of the *B. porcorum* QD2021

Type	Value	% of total
Genome size (bp)	2,271,736	100%
%GC content of genome	38.51%	
Gene number	2,578	100%
Gene total length (bp)	1,991,115	87.65%
Gene average length (bp)	772	
No. of plasmid	1 (70,040 bp)	3.08%
%GC content of plasmid	32.84%	
16S rRNA genes	3 (4,549 bp)	0.12%
23S rRNA genes	3 (8,241 bp)	0.12%
5S rRNA genes	3 (321 bp)	0.12%
tRNA genes	42 (3,246 bp)	1.63%
Genome islands	4 (53,686 bp)	2.36%
Prophage	5 (275,919 bp)	12.15%
CRISPRs	3 (8,911 bp)	0.39%
LTR	67 (4,971 bp)	0.22%
DNA transposon	16 (958 bp)	0.04%
LINEs	21 (1,356 bp)	0.06%
SINEs	15 (947 bp)	0.04%
Rolling circle	2 (118 bp)	0.01%
TR	319 (30,528 bp)	1.34%
Minisatellite DNA	223 (12,402 bp)	0.55%
Microsatellite DNA	2 (94 bp)	0.00%

There were 121 scattered repeat sequences in the full genome of QD2021, including 67 long terminal repeats (LTRs), 21 long interspersed repeats (LINEs), 16 transposons, 15 short interspersed repeats (SINEs), and 2 rolling circles. Moreover, 319 tandem repeat sequences were predicted, including 223 minisatellite DNAs and 2 microsatellite DNAs (Table 2).

**Fig. 4:**
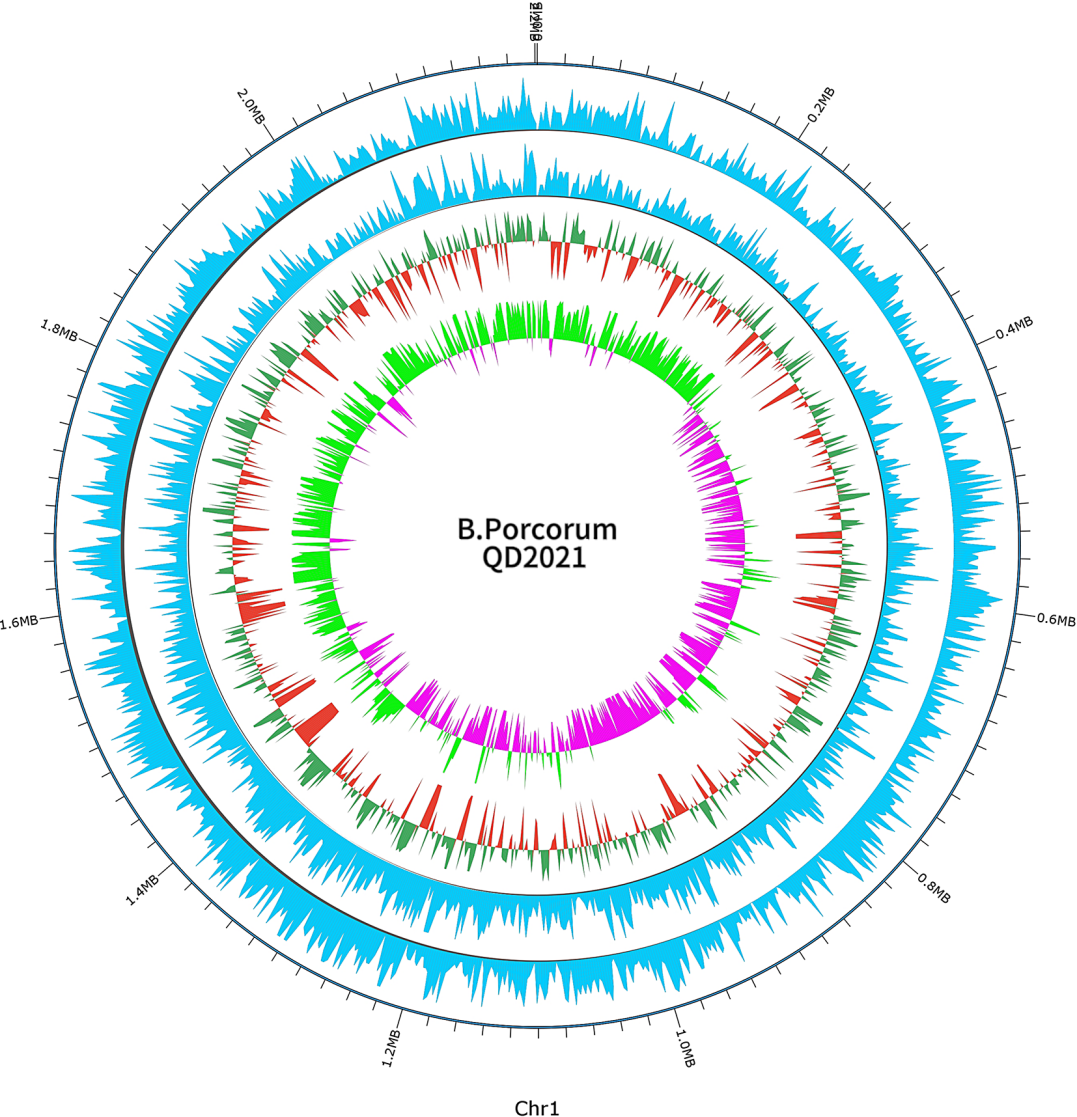
Distribution map of the *B. porcorum* QD2021 epigenetic modification. From outside to center, the results correspond to genome positions in kb (ring 1), modifications in the sense strand (ring 2), modifications in the antisense strand (ring 3), and GC content and GC deviations from the average (ring 4)

In addition, 4 GIs (2.36% of 53,686 bp), 5 prophages (12.15% of 275,919 bp), and 3 CRISPRs (0.39% of 8,911 bp) located on chromosomes were predicted (Table 2, Additional file 1). Additionally, the methylation data of the *B. porcorum* QD2021 whole genome are listed in Fig. 4 (Additional file 1).

### Gene functional analysis of *B. Porcorum* QD2021

The most abundant gene functions were predicted by NR (1,858/2,578), followed by GO (1,459/2,578), Pfam (1,459/2,578), COG (1,357/2,578), KEGG (1,225/2,578), Swiss-Port (630/2,578), TCDB (92/2,578), and CAZy (77/2,578).

**Fig. 5:**
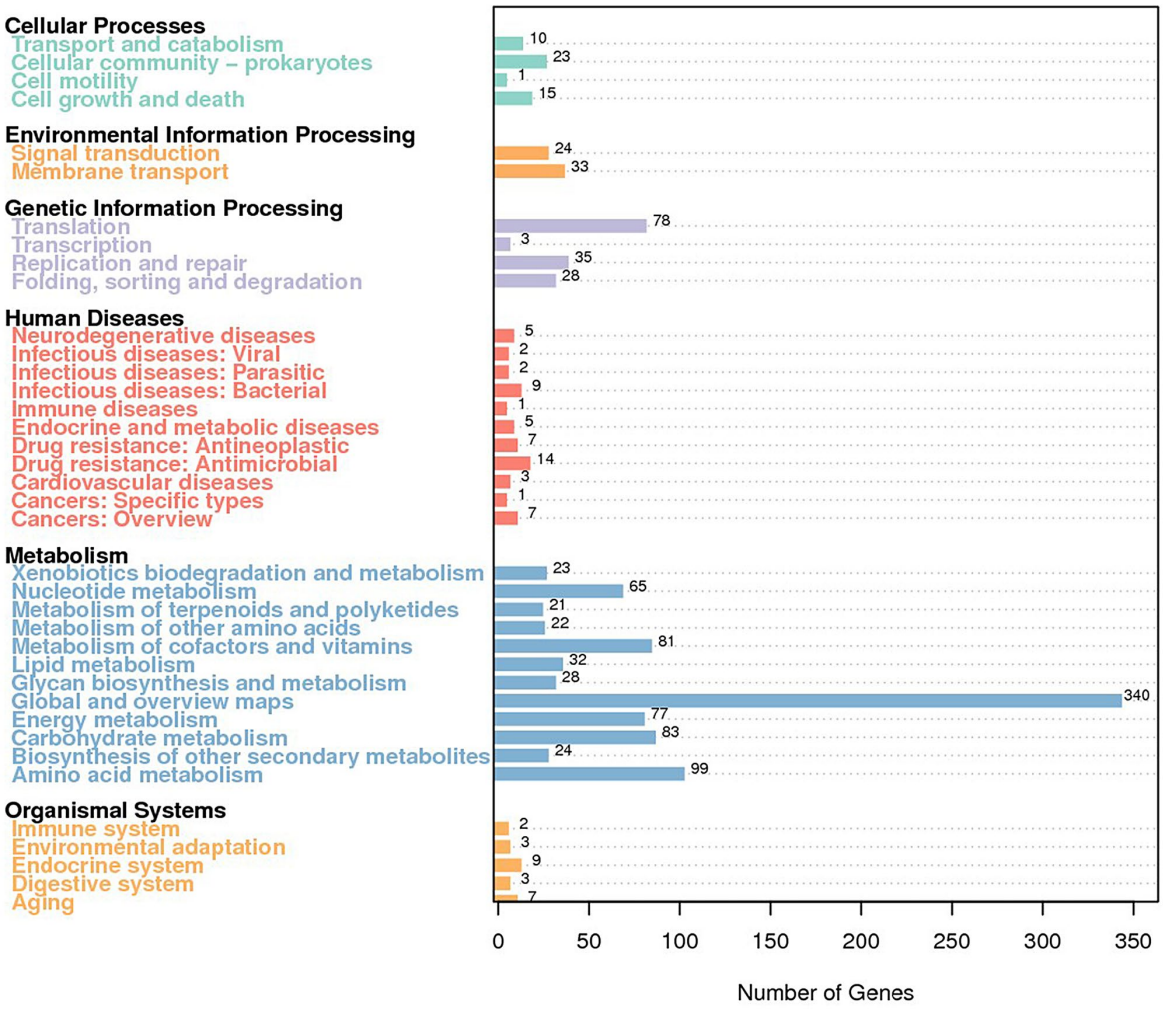
Bacterial gene functional annotation of the KEGG metabolic pathway

The 1,225 KEGG-annotated genes were distributed into 6 categories (Fig. 5, Additional file 2). Among them, the most populated class was represented by metabolism pathways (895), followed by genetic information processing (144), environmental information processing (57), human diseases (56), cellular processes (49), and organismal systems (24). The most abundant class was the global and overview maps from the “Metabolism” category (340 genes), followed by amino acid metabolism (99 genes) and carbohydrate metabolism (88 genes) (Fig. 5).

**Fig. 6:**
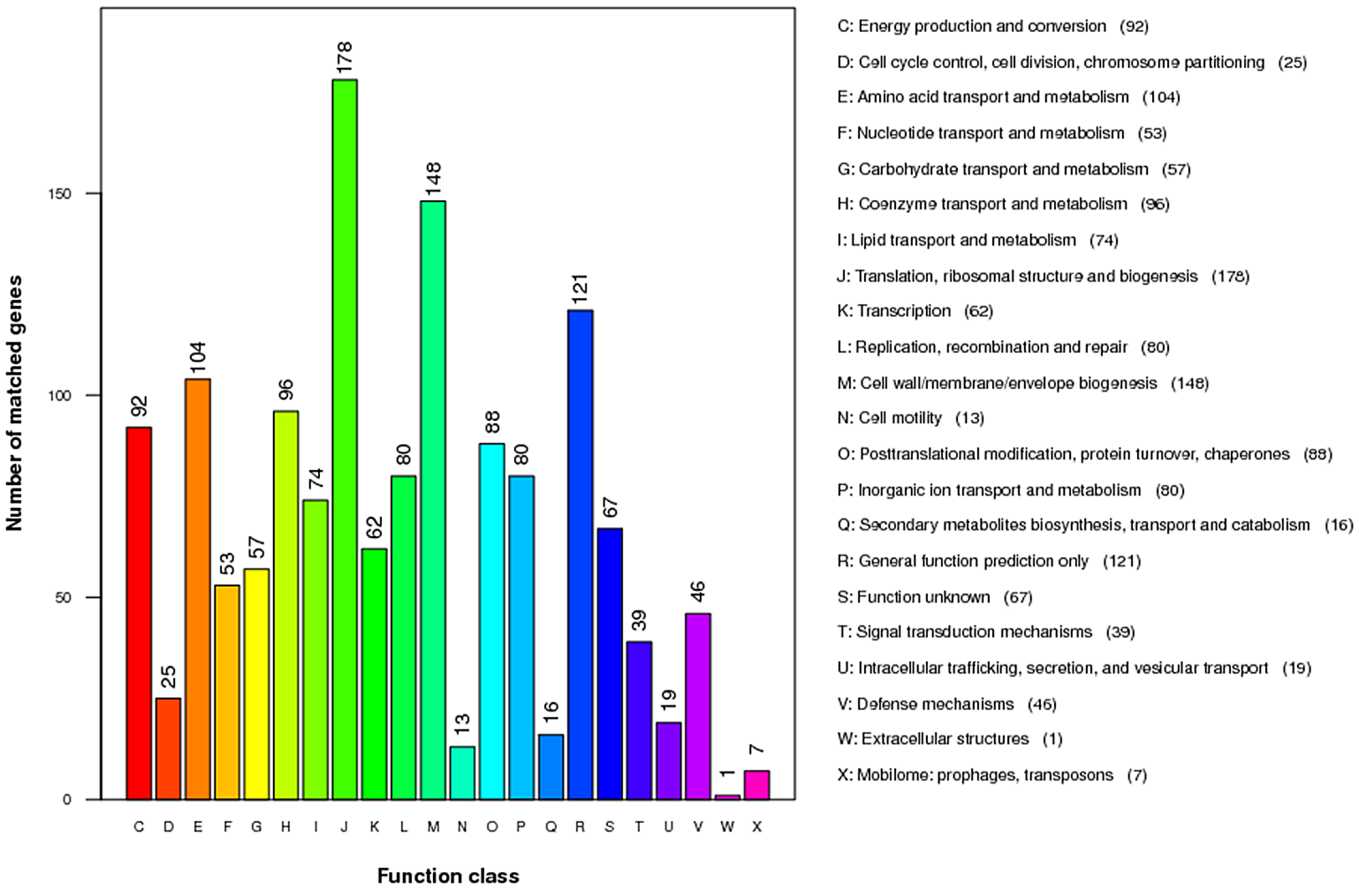
The genes of the *B. porcorum* QD2021 genome in COG functional categories

In addition, 56.87% (1,357/2,578) of the protein-coding genes were assigned 1,466 putative functions in the COG database (Fig. 6, Additional file 2). Among them, 1,259 protein-coding genes were assigned with one signal function, and the remaining 98 protein-coding genes were assigned to two to three putative functions. According to the COG categorization, “translation”, “ribosomal structure” and “biogenesis” were the most enriched functions (178 genes), followed by “cell wall/membrane/envelope biogenesis” (148 genes), “general function prediction only” (121 genes), “amino acid transport and metabolism” (104 genes), and “coenzyme transport and metabolism” (96 genes). Furthermore, this study identified a total of 67 hypothetical genes, which may need further exploration.

**Fig. 7:**
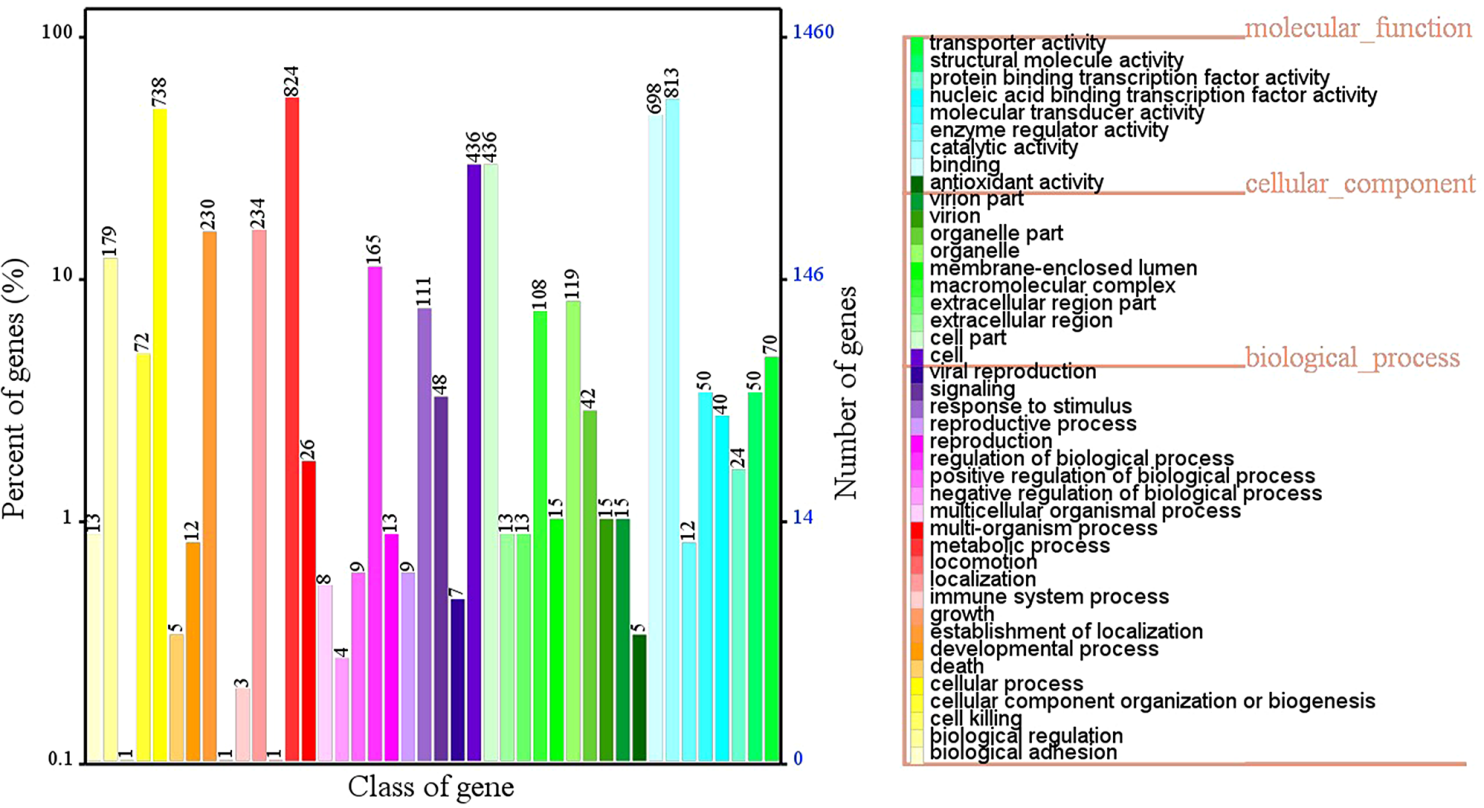
GO classification of bacterial gene function annotation

According to the GO analysis, a total of 1,459 protein-encoding genes categorized into biological process, cellular component, and molecular function categories were annotated (Fig. 7, Additional file 2). The top two annotated molecular functions were catalytic activity (813 genes) and binding (698 genes). The cell (436 genes), cell part (436 genes), and organelle (109 genes) were the top three enriched cellular components. A total of 824 and 738 genes were enriched in the molecular functions metabolic process and development process, respectively.

**Fig. 8:**
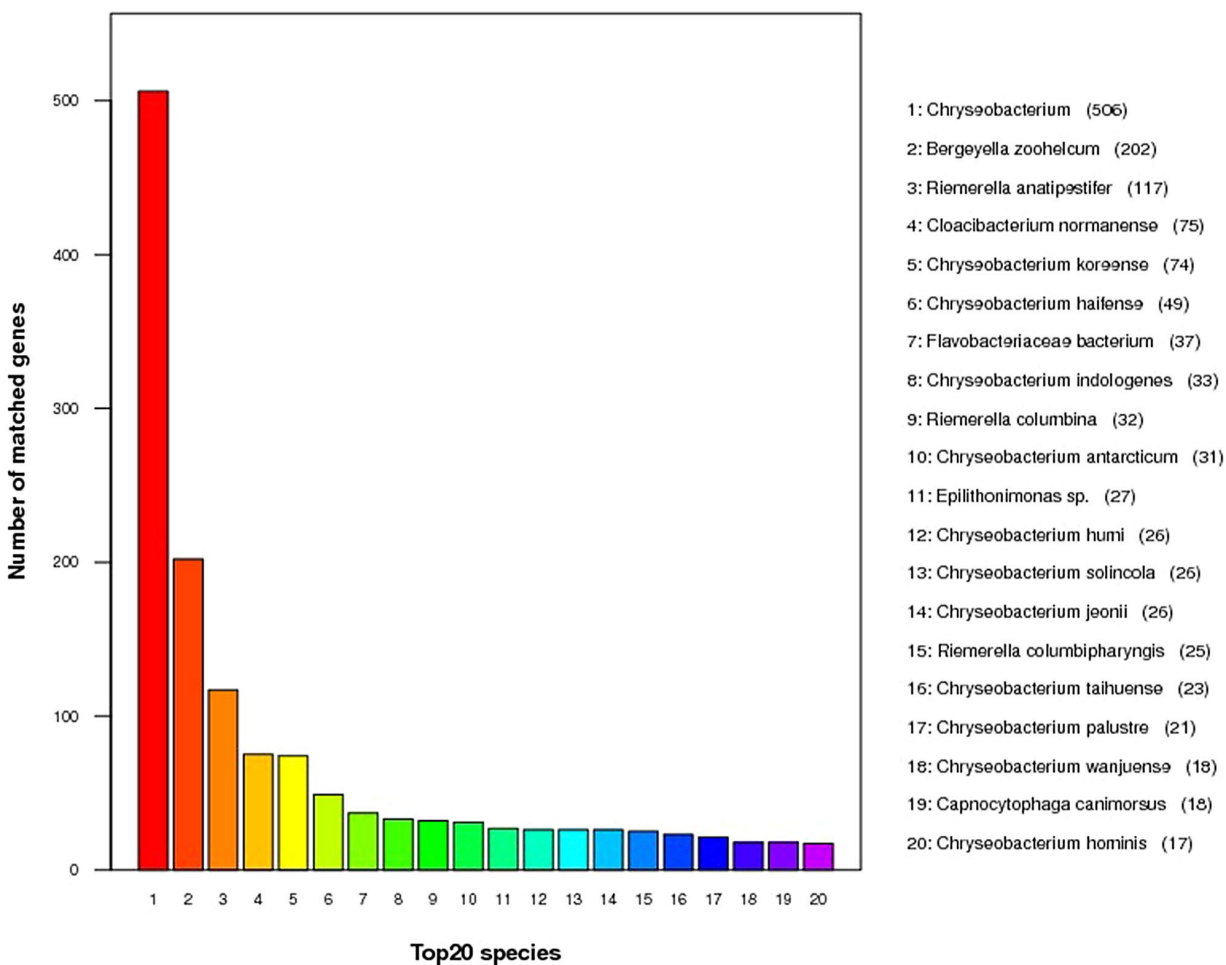
Annotated species statistics of the NR database (top 20 species)

A total of 1,858 genes within 80 different bacterial species were annotated in the NR database (Fig. 8, Additional file 2). Among the top 20 species, 506 genes were associated with *Chryseobacterium*, 202 genes were associated with *B. zoohelicum* species, and 117 genes were associated with *Rimerella anatipesdtifer*.

### Pathogenic analysis of *B. Porcorum* QD2021

The CARD, ARDB, VFDB and PHI databases were used to identify genes related to antibiotic resistance and virulence factors in the genome of *B. porcorum* QD2021. According to the ARDB analysis, 10 genes were annotated, 4 of which were related to tetracycline resistance; 2 of which were macrolide-lincosamide-streptogramin_B (MLS_B_); and 1 each of cephalosporin, aminoglycoside, bacitracin, and chloramphenicol resistance (Additional file 3). According to the CARD analysis, 20 genes were annotated, among which *tetX* was the most prevalent (6/20), and one each of *adeC*, *TriC*, *mfd*, *adeG*, aminocoumarin-resistant *alaS*, *floR*, elfamycin-resistant EF-Tu, *gyrA*, *ROB-1*, *rpoB*, *ileS*, *katG*, *APH*(*3’*)-*Ia* and *OXA*-*368* were the most common genes (Additional file 1). Moreover, 126 genes categorized into six classes were shown to be involved in bacteria-host reactions according to the PHI analysis. The most populated class was “reduced virulence” (80 genes), followed by “unaffected pathogenicity” (22 genes) and “hypervirulence” (7 genes) (Additional file 3). The VFDB inferred a total of 65 genes, most of which were related to virulence factors, such as “capsule,” “LPS,” and “Urease” (Additional file 3).

### Comparative genomic analysis of *B. Porcorum* QD2021

The *is*DDH and ANI values of the isolated strain *B. porcorum* QD2021 were compared with those of 5 *B. zoohelicum* strains and 3 *B. cardium* strains. The *is*DDH values of *B. zoohelcum* ATCC43767 (22.30%), *B. zoohelcum* CCUG30536 (21.90%), *B. zoohelcum* NCTC11660 (22.50%), *B. zoohelcum* NCTC11661 (22.10%), *B. zoohelcum* NCTC12929 (21.90%), *B. cardium* HPQL (22.00%), *B. cardium* SRR1044034 (19.50%), and *B. cardium* strain 1 (22.10%) were all less than the 70% cutoff points recommended for delineating species. Moreover, the ANI values of *B. zoohelcum* ATCC43767 (73.12%), *B. zoohelcum* CCUG30536 (72.85%), *B. zoohelcum* NCTC11660 (73.13%), *B. zoohelcum* NCTC11661 (73.04%), *B. zoohelcum* NCTC12929 (72.95%), *B. cardium* HPQL (71.23%), *B. cardium* SRR1044034 (71.23%), and *B. cardium* strain 1 (71.59%) were also less than the 95–96% cutoff points, which indicated that the isolated strain *B. porcorum* QD2021 was a distinct species of *Bergeyella* spp.

**Fig. 9:**
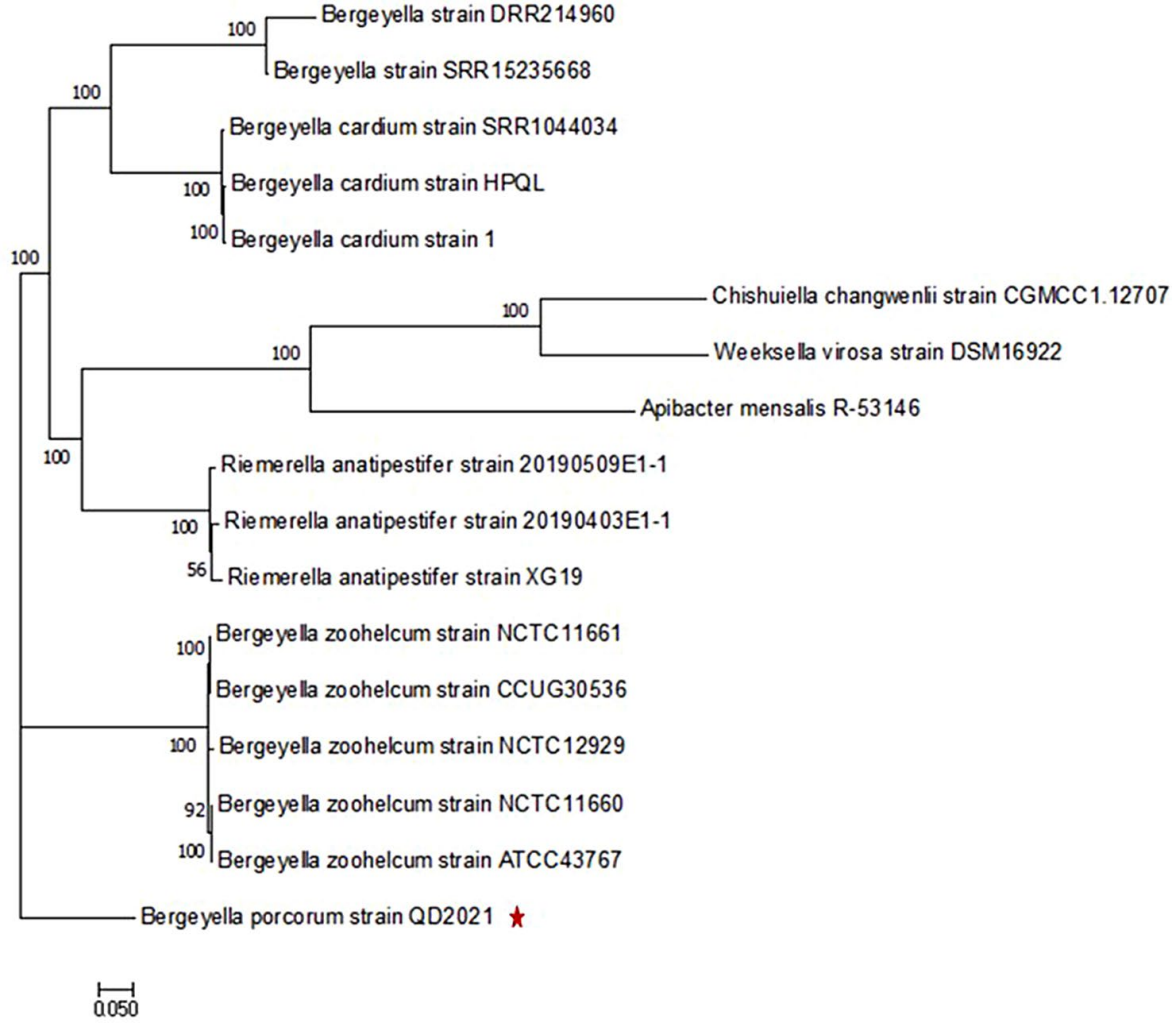
Phylogenetic and comparative genomic analysis of *B. porcorum* QD2021. A total of 16 complete genomes were analyzed, and an unrooted phylogenetic tree was inferred by the maximum likelihood (ML) method with RAxML. The scale bar indicates 0.05 nucleotide substitution per nucleotide position

To further explore the evolutionary relationships, the whole genome of *B. porcorum* QD2021 was compared with those of 10 other strains of the genus *Bergeyella* and 6 strains of other relative genus for phylogenetic analysis. The results showed that the 3 *B. cardium* strains (SRR1044034, 1 and HPQL) formed a clade with the 2 *Bergeyella* strains (DRR214960 and SRR15235668), indicating their genetic relatedness. The three *Riemerella anatipestifer* strains (XG19, 20190509E1-1 and 20190403E1-1) formed a clade with *Apibacter mensalis* R-53146, *Weeksella cirosa* DSM16922, and *Chishuiella changwenlii* CGMCC1.12707. The five *B. zoohelcum* strains (CCUG30536, NCTC11660, NCTC11661, NCTC12929 and ATCC43767) formed a clade (Fig. 9). Additionally, *B. porcorum* QD2021 formed a separate clade, demonstrating that the isolated strain is a new species belonging to the genus *Bergeyella*.

## Discussion

*Bergeyella* spp. (previously known as *Weeksella*) are part of the normal oral microbiota of animals such as cats and dogs [39], but these bacteria are not well characterized. The genus *Bergeyella* is a rarely reported zoonotic pathogen, with *B. zoohelcum* being the only well-described zoonotic pathogen affecting humans [4, 40]. To date, most of the reported infections have been related to bites from dogs [41, 42], cats [4], Siberian tigers [43], or contact with these animals [40]. *B. cardium* was first isolated from IE patients in 2015 and described as a new species of the *Bergeyella* genus that has not yet been validated [2, 44]. Recently, 4 cases of *B. cardium* have been isolated worldwide from patients with infective endocarditis [2, 44, 45]. *B. porcorum* was first isolated from pigs in 2016, but its relationship with swine pneumonia is still ambiguous [3, 46]. Currently, the whole-genome sequences of *B. zoohelcum* and *B. cardium* have been released, but the whole-genome sequence of *B. porcorum* has not yet been obtained.

16S rRNA gene sequencing analysis is a routinely used method for identifying poorly described or phenotypically aberrant isolates; this method provides unambiguous data even for rare isolated strains and can lead to the identification of novel pathogens [47]. In this study, the isolates we identified were closely related to *B. porcorum* in terms of both their similar pathogenicity and phylogenetic relationship. This study showed that the 16S rRNA gene of the isolates exhibited the highest homology with that of *B. porcorum* but fairly low similarity to that of *B. zoohelcum* and *B. porcorum*.

ANI and isDDH are the traditional “gold standards” for circumscribing a bacterial species, and pairwise comparisons of strains with ANI values ≥ 95% and isDDH values ≥ 70% are typically considered to indicate the same species [48]. In this study, lower ANI and isDDH values were detected between the isolated strain and *B. zoohelcum*, and between the isolated strain and *B. cardium* strains, which is consistent with the findings of the phylogenetic analyses and indicates that the isolated strain belongs to *B. porcorum*.

Nevertheless, few studies have examined *B. porcorum*; thus, no comparable genomic information, such as reference sequences, GC contents, or repetitive sequences, is available. Herein, we performed whole-genome sequencing of the newly isolated *B. porcorum* sp. by combining second- and third-generation sequencing data. Such a genome assembly can reverse and decrease the interference of abnormal GC contents, high repetition and hybridity, thus improving the integrity and uniformity of the generated genome sequence [17].

The presence of prophages allows some bacteria to acquire antibiotic resistance, enhances environmental adaptability, and improves adhesion [49]. In *B. porcorum* QD2021, the detection of five prophages may enhance its ability to produce genetic exchange among microflora. In addition, four genomic islands were detected in *B. porcorum*, which may contribute to its pathogenicity.

## Conclusions

In this study, we report the morphological, physiological, and genomic characteristics of a newly identified *B. porcorum* sp. nov. strain isolated from a pig. To our knowledge, this is the first complete genome sequencing study performed on *B. porcorum* to provide fundamental information to better understand *B. porcorum*. The high-quality genome obtained in this study could serve as a valuable genomic resource for future research on *B. porcorum.*

### Electronic supplementary material

Below is the link to the electronic supplementary material.

Supplementary Material 1

Supplementary Material 2

Supplementary Material 3

## Data Availability

The 16S rRNA sequence and assembled genome sequence of *Bergeyella porcorum* sp. nov. has been provided to the NCBI Sequence Read Archive under accession numbers OR493469 and PRJNA1021403, respectively, and all the raw data are available. The scripts for performing bioinformatics analyses and supplementary files in this work can be found in GitHub at https://github.com/Lg890810/Complete-Genome-sequence-of-B.-porcorum.
